# Ubiquitination of Sec22b by a novel *Legionella pneumophila* ubiquitin E3 ligase

**DOI:** 10.1128/mbio.02382-23

**Published:** 2023-10-26

**Authors:** Kelong Ma, Rundong Shu, Hongtao Liu, Jiaqi Fu, Zhao-Qing Luo, Jiazhang Qiu

**Affiliations:** 1 State Key Laboratory for Diagnosis and Treatment of Severe Zoonotic Infectious Diseases, Key Laboratory for Zoonosis Research of the Ministry of Education, College of Veterinary Medicine, Jilin University, Changchun, China; 2 Center for Pathogen Biology and Infectious Diseases, The First Hospital of Jilin University, Changchun, China; 3 Purdue Institute for Inflammation, Immunology and Infectious Disease and Department of Biological Sciences, Purdue University, West Lafayette, Indiana, USA; Institut Pasteur, Paris, France

**Keywords:** Legionella, effector protein, ubiquitination, E3 ligase, Sec22b

## Abstract

*Legionella pneumophila* is a facultative intracellular pathogen that causes legionellosis. The key to its virulence is the delivery of hundreds of effector proteins into host cells via the defective in organelle trafficking/intracellular multiplication type IV secretion system. These effectors modulate numerous host signaling pathways to create a niche called the *Legionella*-containing vacuole (LCV) permissive for its intracellular replication. Previous investigation revealed that exploitation of the host ubiquitin system is among the most important strategies used by *L. pneumophila* to coopt host processes for its benefit. Here, we show that the effector *Legionella* ubiquitin ligase gene 15 (Lug15) (Lpg2327), which has no detectable homology with any enzyme involved in ubiquitin signaling, is an E3 ligase. In *L. pneumophila*-infected cells, Lug15 is localized on the LCV and impacts its association with polyubiquitinated proteins. We also demonstrate that Sec22b is ubiquitinated and recruited to the LCV by Lug15. Thus, our results establish Lug15 as a novel E3 ligase that functions to recruit a SNARE protein to remodel the *L. pneumophila* phagosome.

**IMPORTANCE:**

Protein ubiquitination is one of the most important post-translational modifications that plays critical roles in the regulation of a wide range of eukaryotic signaling pathways. Many successful intracellular bacterial pathogens can hijack host ubiquitination machinery through the action of effector proteins that are injected into host cells by secretion systems. *Legionella pneumophila* is the etiological agent of legionellosis that is able to survive and replicate in various host cells. The defective in organelle trafficking (Dot)/intracellular multiplication (Icm) type IV secretion system of *L. pneumophila* injects over 330 effectors into infected cells to create an optimal environment permissive for its intracellular proliferation. To date, at least 26 Dot/Icm substrates have been shown to manipulate ubiquitin signaling via diverse mechanisms. Among these, 14 are E3 ligases that either cooperate with host E1 and E2 enzymes or adopt E1/E2-independent catalytic mechanisms. In the present study, we demonstrate that the *L. pneumophila* effector *Legionella* ubiquitin ligase gene 15 (Lug15) is a novel ubiquitin E3 ligase. Lug15 is involved in the remodeling of LCV with polyubiquitinated species. Moreover, Lug15 catalyzes the ubiquitination of host SNARE protein Sec22b and mediates its recruitment to the LCV. Ubiquitination of Sec22b by Lug15 promotes its noncanonical pairing with plasma membrane-derived syntaxins (e.g., Stx3). Our study further reveals the complexity of strategies utilized by *L. pneumophila* to interfere with host functions by hijacking host ubiquitin signaling.

## INTRODUCTION

*Legionella pneumophila* is a Gram-negative opportunistic pathogen that is ubiquitous in aquatic environments, where it parasitizes freshwater protozoa, including amoebae (1). Inhalation of aerosols containing *L. pneumophila* by susceptible individuals often causes legionellosis, which has two clinical forms: Legionnaires’ disease, a potentially fatal form of pneumonia, and Pontiac fever, a mild influenza-like illness (2). Once *L. pneumophila* is engulfed by phagocytes, the bacterial phagosome bypasses the endocytic maturation process and thus resists lysosomal degradation. Instead, the phagosome is remodeled by vesicles of the secretory, endocytic, and retrograde trafficking pathways of the host cell (3–5), resulting in its conversion into an ER-like organelle called the *Legionella*-containing vacuole (LCV).

The biogenesis and maturation of the LCV require the defective in organelle trafficking (Dot)/intracellular multiplication (Icm) type IV secretion system, which injects approximately 330 effectors into the cytosol of infected cells (6). These effectors function to modulate various host cell processes, such as membrane trafficking, autophagy, and immune responses, by targeting essential regulatory proteins with distinct biochemical activities (6). For example, the small GTPase Rab1, which is essential for vesicle transport between the ER and the Golgi apparatus, is extensively targeted and modified by multiple Dot/Icm effectors via distinct mechanisms. Such manipulation is required for the recruitment of ER-derived vesicles to the LCV (7). Upon being recruited to the LCV by the multifunctional effector SidM/DrrA, Rab1 is first activated via the GEF activity of SidM/DrrA (8, 9) followed by the GAP activity of LepB (10). Moreover, Rab1 activity is regulated by several effector-catalyzed post-translational modifications (PTMs) at different stages of infection. These PTMs include reversible AMPylation mediated by SidM/DrrA (11) and SidD (12, 13), reversible phosphorylcholination executed by AnkX and Lem3 (14, 15), reversible phosphoribosyl-linked serine ubiquitination catalyzed by SidEs (16, 17), DupA and DupB (18, 19), lysine-linked ubiquitination mediated by SidC/SdcA (20), and glucosylation induced by SetA (21). These processes highlight the sophisticated strategies adopted by *L. pneumophila* to subvert vesicle trafficking. Other small GTPases, including members of the Arf (22), Ran (23), and Rap (24) families and the large GTPase DNM1L (25), are also targeted by specific Dot/Icm effectors to promote LCV formation.

Intracellular membrane fusion is mediated by specific pairing of soluble N-ethylmaleimide-sensitive factor attachment protein receptors (SNAREs) on the vesicle membrane and targeting organelle (26). Soon after bacterial entry into the host cell, the vesicular SNARE (v-SNARE) Sec22b, which is involved in the targeting and fusion of ER-derived vesicles to the Golgi apparatus, is recruited to the bacterial phagosome, despite the absence of its cognate target SNAREs (t-SNAREs) Stx5, rBet1, and membrin (27, 28) on this organelle. Instead, noncanonical pairing between Sec22b and plasma membrane (PM)-derived t-SNAREs triggers the fusion between ER-originated vesicles and the LCV (7, 29).

The LCV is coated with polyubiquitinated proteins in a Dot/Icm-dependent manner to facilitate optimal intracellular bacterial growth (30). Consistent with this observation, multiple effector proteins with ubiquitin ligase activity have been found to be associated with the LCV (31). Although the host targets of most of these bacterial ubiquitin ligases remain unknown, several have been shown to play a role in the recruitment of polyubiquitinated proteins to the LCV (32, 33). For example, the small GTPase Rab10 is ubiquitinated and recruited to the LCV by members of the SidC family and is required for optimal intracellular bacterial growth (34), consistent with the observation that bacterial vacuoles harboring mutants lacking *sidC* and *sdcA* are decorated with fewer ubiquitin (Ub) signals (32). In addition to E3 ubiquitin ligases, the *L. pneumophila* genome also codes for deubiquitinases (DUBs) that recognize distinct chain types (6). Apparently, some of these DUBs and E3 ligases function coordinately to coopt host ubiquitin signaling. For instance, we previously demonstrated that the DUB Lem27 functions to regulate protein ubiquitination on the LCV by antagonizing the activity of SidC and SdcA (35).

Here, we demonstrate that the Dot/Icm substrate Lpg2327 [*Legionella* ubiquitin ligase gene 15 (Lug15)] is an E3 ubiquitin ligase critical for the recruitment of polyubiquitin conjugates, including the SNARE protein Sec22b, to the bacterial phagosome.

## RESULTS

### Lpg2327 is an E3 ubiquitin ligase

**Fig 1:**
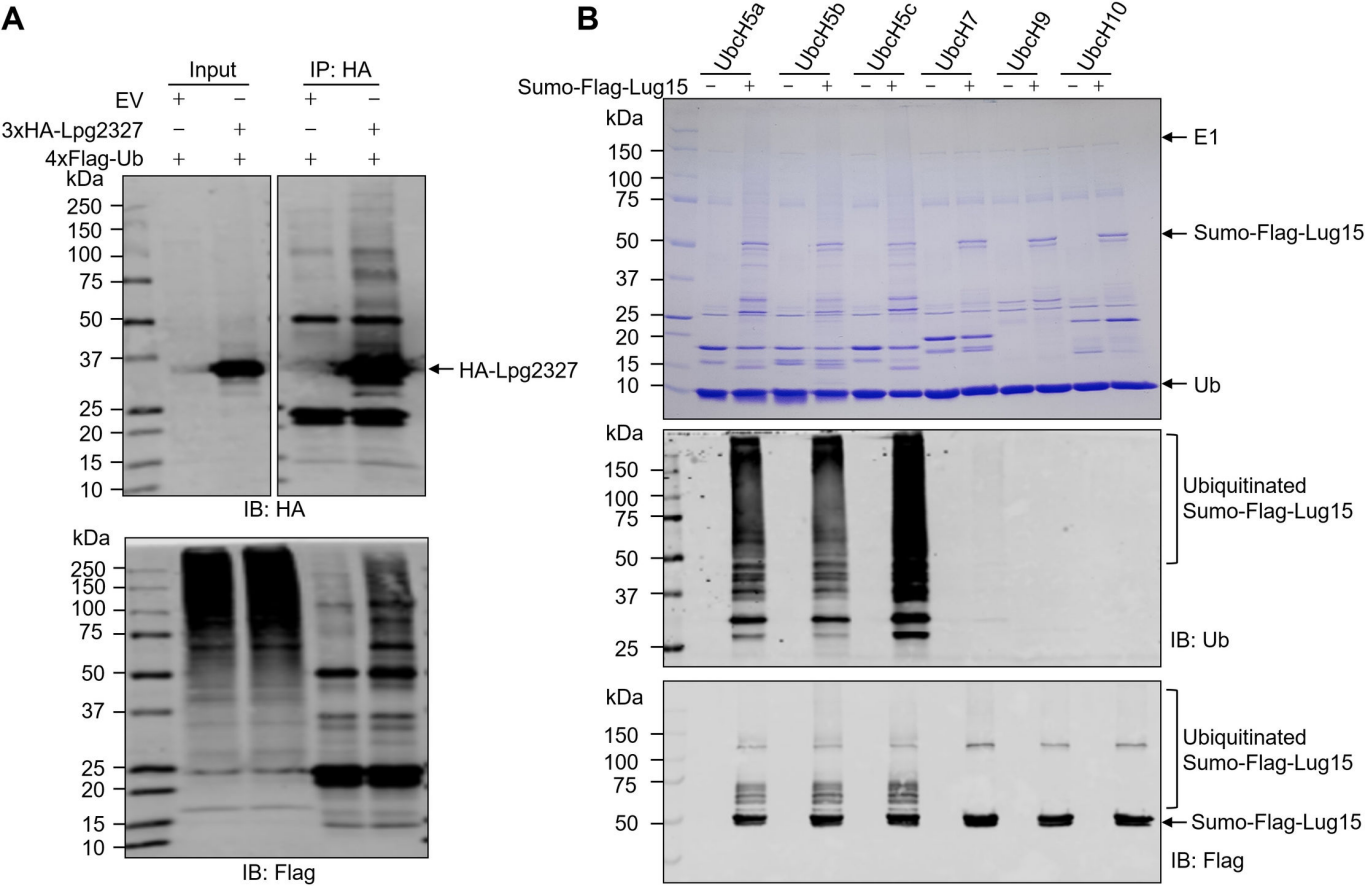
Lpg2327 possesses E3 ubiquitin ligase activity. (**A**) Lpg2327 is ubiquitinated when ectopically expressed in mammalian cells. HEK239T cells were cotransfected with plasmids expressing 3×HA-Lpg2327 and 4×Flag-Ub. After immunoprecipitation of the cell lysates with anti-HA agarose, 3×HA-Lpg2327 bound to the beads was detected by immunoblotting with anti-HA and anti-Flag antibodies. (**B**) *In vitro* ubiquitination assay. Recombinant Sumo-Flag-Lug15 (Lpg2327) was incubated with E1 and a panel of mammalian E2s in a buffer containing Ub, Mg^2+^, and ATP. After incubation at 37°C for 2 h, proteins in the reactions were separated by sulfate-polyacrylamide gel electrophoresis (SDS-PAGE). Polyubiquitination of Lug15 was visualized by Coomassie brilliant blue (CBB) staining (top) or detected by immunoblotting with anti-Ub (middle) and anti-Flag (bottom) antibodies. The data shown in (**A**) and (**B**) are from one representative of three independent experiments.

Lpg2327 is a Dot/Icm substrate containing 297 amino acids (36). As bioinformatics analysis of Lpg2327 using pairwise comparison of profile hidden Markov models (with HHpred) (37) did not retrieve any meaningful hits, we turned to employ immunoprecipitation followed by mass spectrometry analysis to investigate its host-interacting partners. Detection of ectopically expressed 3×HA-tagged Lpg2327 in HEK293T cells with an HA-specific antibody resulted in extensive smeared signals with molecular weights higher than that of 3×HA-Lpg2327, a pattern often associated with ubiquitinated proteins (Fig. S1). We thus coexpressed 3×HA-Lpg2327 with 4×Flag-Ub and detected the protein obtained by immunoprecipitation with an anti-Flag antibody, which revealed similar signals (Fig. 1A), suggesting that Lpg2327 either is an ubiquitin E3 ligase that undergoes extensive self-modification or is extensively ubiquitinated by host enzymes. Because of its potential E3 ligase activity, we designated Lpg2327 as Lug15.

**Fig 2:**
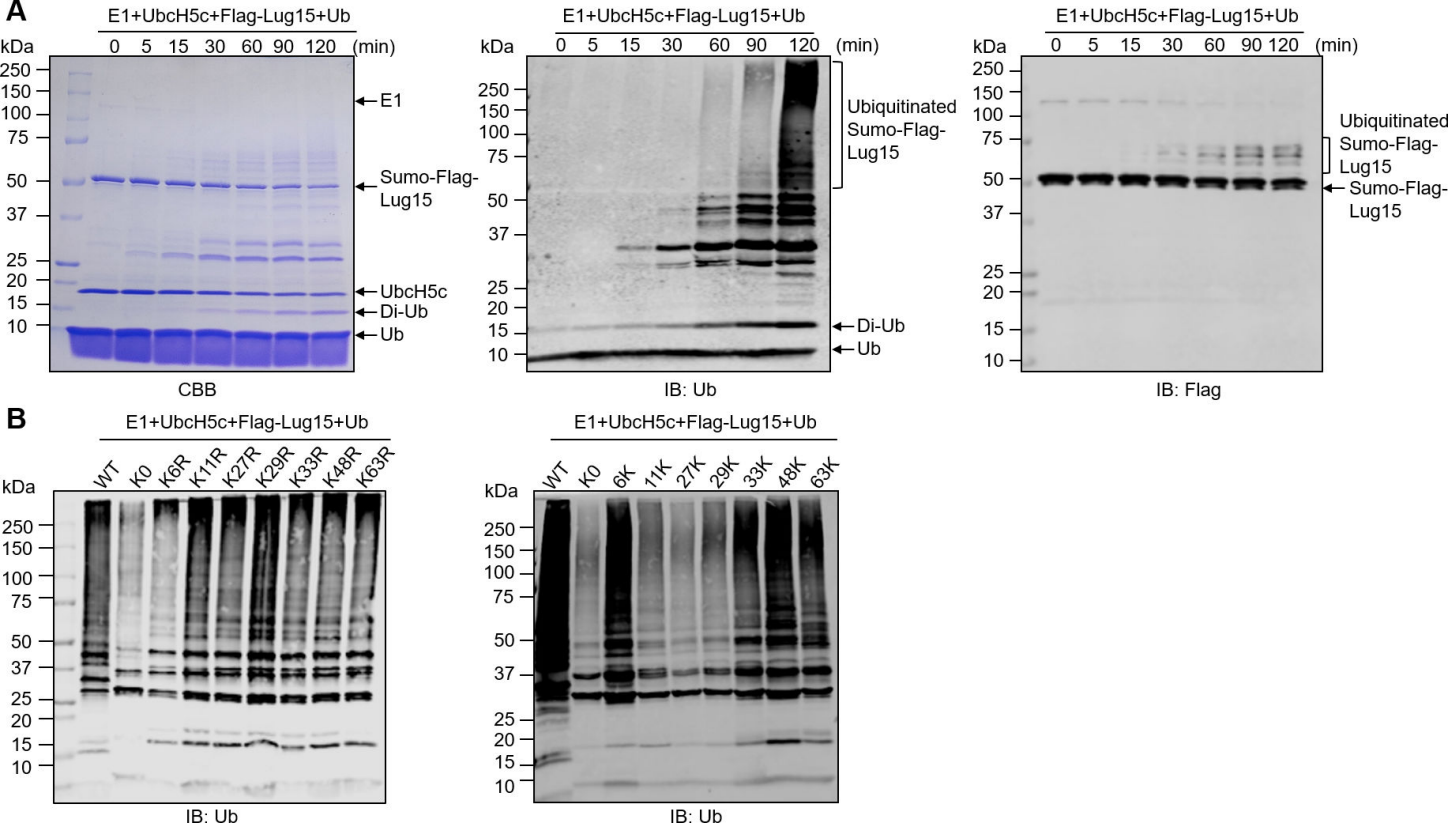
Time course and linkage preference of Lug15-induced ubiquitination. (**A**) Time-dependent ubiquitination assay. The reaction mixture consisting of E1, UbcH5C, Ub, and Lug15 was incubated at 37°C. At the indicated times, the reaction in obtained aliquots was terminated by the addition of 5×SDS sample buffer. The formation of polyubiquitinated species was analyzed by CBB staining (left) or by immunoblotting using anti-Ub (middle) and anti-Flag (right) antibodies. (**B**) Ubiquitin linkage preference of Lug15. *In vitro* ubiquitination assays were set up using Lug15, E1, UbcH5C, and a series of ubiquitin mutants with a single K-to-R mutation (left) or with only one available lysine residue (right). K0 represents the ubiquitin mutant lacking all seven lysine residues. Reactions were allowed to proceed for 2 h at 37°C. Protein ubiquitination was detected by immunoblotting with an antibody specific for ubiquitin. The data are from one representative of three independent experiments.

To distinguish between these two possibilities, we purified recombinant Lug15 and performed biochemical assays using several E2 enzymes with autoubiquitination as a readout. Strikingly, characteristic ubiquitination signals were observed in reactions containing UbcH5 family E2s (Fig. 1B). Signals were also detected in reactions using UbcH7 but had a considerably lower intensity (Fig. 1B). In contrast, autoubiquitinated Lug15 was not detectable in reactions containing UbcH9 or UbcH10 (Fig. 1B). Consistent with the robust ligase activity with UbcH5, Lug15 directly interacts with this E2 enzyme (Fig. S2). The activity of Lug15 requires all components of the canonical ubiquitination machinery, as autoubiquitination occurred only in reactions containing E1, E2, Ub, ATP, and Mg^2+^ (Fig. S3). Time kinetics experiments showed the gradual appearance of high-molecular weight ubiquitinated proteins accompanied by a decrease in the amount of unmodified Lug15 as the reaction proceeded (Fig. 2A). Taken together, these results establish that Lug15 functions as an E3 ubiquitin ligase.

### Lug15 catalyzes the formation of multiple types of ubiquitin chain linkages

Seven lysine residues (K6, K11, K27, K29, K33, K48, and K63) and the primary methionine residue (M1) in ubiquitin can participate in the extension of ubiquitin chains, resulting in the formation of eight homotypic polyubiquitin chains with a single linkage type or multiple complex heterotypic chains that contain several linkage types (38). To investigate the features of the polyubiquitinated products assembled by Lug15, we utilized a panel of ubiquitin mutants, each with mutation of one lysine residue to arginine (K to R), in the *in vitro* ubiquitination reaction. Although the amounts varied, all the ubiquitin single K-to-R mutants could still be utilized by Lug15 to form polyubiquitinated products (Fig. 2B). As expected, the ubiquitin mutant in which each of the seven Ks was replaced with R (K0) lost the ability to form polyubiquitin chains (Fig. 2B). These results indicate that Lug15 can catalyze the formation of ubiquitin chains with each type of lysine linkage. We further assayed the linkage preference of Lug15 with ubiquitin derivatives containing only one lysine. Polyubiquitin chain synthesis was markedly impaired when Ub-11K, Ub-27K, or Ub-29K was used (Fig. 2B). In contrast, although less effective than wild-type (WT) ubiquitin, reactions containing Ub-6K, Ub-33K, Ub-48K, or Ub-63K produced considerable amounts of polyubiquitinated species (Fig. 2B). Collectively, these results demonstrate that Lug15 can utilize each of the seven lysine residues in ubiquitin to assemble polyubiquitin chains, with a preference for the K6, K33, K48, and K63 linkage types. In support of this, western blot analysis of these ubiquitination samples with linkage-specific antibodies showed extensive formation of K48- and K63-linked polyubiquitin conjugates assembled by Lug15 (Fig. S4).

### A central region of Lug15 is required for its E3 ligase activity

**Fig 3:**
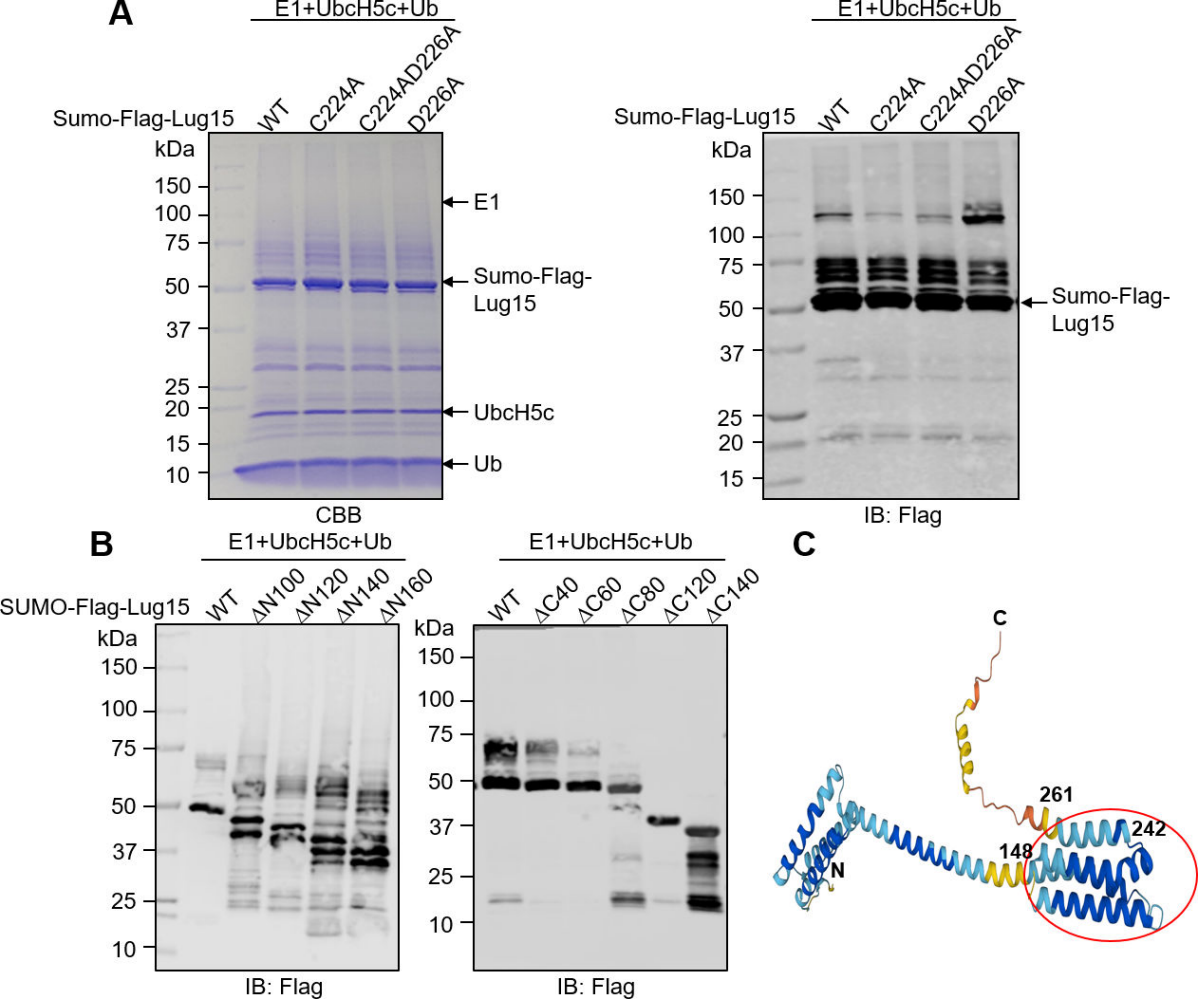
The central region of Lug15 mediates its E3 ligase activity. *In vitro* ubiquitination assays were performed using the indicated Lug15 CxD mutants (**A**) or truncation mutants (**B**) in the presence of E1, UbcH5c, and Ub. After incubation at 37°C for 2 h, the reaction mixtures were separated by SDS-PAGE, and ubiquitin conjugates were detected by CBB staining or immunoblotting with an anti-Flag antibody. The data are from one representative of three independent experiments. (**C**) The predicted structure of Lug15 was obtained from the AlphaFold Protein Structure Database. The red circle indicates the potential catalytic domain for the E3 ligase activity of Lug15.

Bioinformatics analysis with programs such HHpred did not reveal any homology between Lug15 and established E3 ligases. In addition, our attempts to solve the structure of Lug15 were not successful. These obstacles have historically hindered us from further elucidating the molecular basis of catalysis induced by Lug15. However, we noted that Lug15 harbors a potential CxD catalytic motif (C_224_xD_226_) found in the E3 ubiquitin ligase effector IpaH3 of *Shigella flexneri* (39). Nevertheless, neither individual nor combined mutation of Cys224 or Asp226 impaired the E3 ligase activity of Lug15 (Fig. 3A), indicating that Lug15 employs a catalytic mechanism different from that of IpaH3. To investigate the catalytic mechanism, we generated a series of Lug15 truncation mutants to determine the region important for its E3 ligase activity. Deletion of 160 residues from its amino terminus (Lug15ΔN160) did not affect the E3 ligase activity of Lug15 (Fig. 3B). However, truncation of 60 amino acids from its carboxyl end (Lug15ΔC60) resulted in a significant reduction in catalysis, while the mutants Lug15ΔC80, ΔC120, and ΔC140 completely abolished its E3 ligase activity (Fig. 3B). Taken together, the catalytic domain of Lug15 for ubiquitination might be located within amino acids ranging from 161 to 237. Importantly, according to the structural information retrieved from the AlphaFold Protein Structure Database (Entry: Q5ZT38) (40, 41), Lug15_161-237_ could form a compact structure that may be critical for enzymatic activity (Fig. 3C).

### Lug15 is expressed throughout the growth cycle and is not essential for intracellular *L. pneumophila* replication in macrophages

*L. pneumophila* adopts a specific biphasic lifecycle that transitions between noninfective replicative and infectious transmissive phases (42). This transition is linked to large-scale transcriptomic alterations mediated by multiple bacterial two-component systems in response to various environmental signals (43). In particular, many effector-encoding genes were found to be expressed in the post-exponential phase in bacteriological media, a profile equivalent to that of the transmissive form of intracellular *L. pneumophila* (44). To obtain insights into the virulence traits of Lug15, we investigated its expression profile in bacteria grown in broth with Lug15-specific antibodies. Interestingly, *L. pneumophila* appeared to produce Lug15 throughout its entire growth cycle, with higher amounts detected in the early exponential phase (Fig. S5). Thus, Lug15 likely plays roles throughout the infection cycle of *L. pneumophila*.

To investigate the role of Lug15 in *L. pneumophila* intracellular proliferation, we constructed an in-frame deletion mutant of Lug15 (Δ*lug15*) and measured its growth in host cells. The growth of the *lug15* deletion mutant was indistinguishable from that of the WT strain in mouse bone marrow-derived macrophages (BMDMs) (Fig. S6). These findings suggest that Lug15 is dispensable for intracellular bacterial growth in this model host.

### Lug15 regulates the accumulation of polyubiquitin species on the LCV

**Fig 4:**
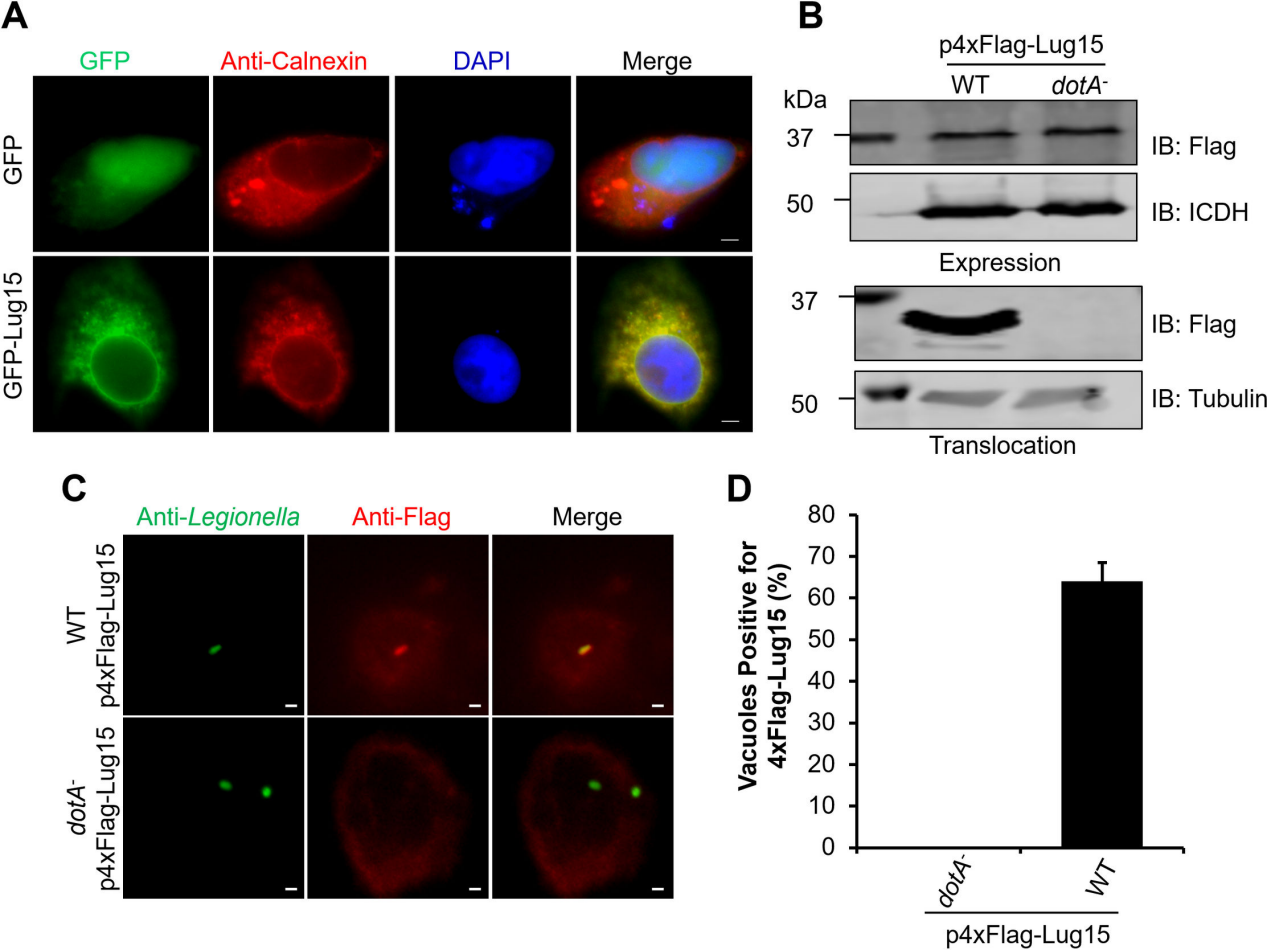
Cellular localization of Lug15. (**A**) Ectopically expressed Lug15 localizes to the ER. HeLa cells were transfected with a plasmid driving the expression of GFP-Lug15. Fixed cells were immunostained with an anti-Calnexin antibody to label the ER. Nuclei were labeled with 4’, 6-diamidino-2-phenylindole (DAPI). Bar, 2 µm. (**B**) Lug15 is delivered into infected host cells. WT and *dotA*^−^ mutant *L. pneumophila* were transformed with a plasmid driving the production of 4×Flag-Lug15. After infection of U937 cells with these strains for 2 h, the cells were lysed with saponin, and the amount of 4×Flag-Lug15 in the cell lysates was measured by immunoblotting with an anti-Flag antibody. Despite its equal expression in both strains (top), 4×Flag-Lug15 was detected only in the saponin-soluble fractions of cells infected with the WT but not the Dot/Icm-deficient *L. pneumophila* strain (bottom). (**C and D**) Lug15 is associated with the LCV during infection. U937 cells were challenged with 4×Flag-Lug15 expressing *L. pneumophila* strains for 2 h. After immunostaining of fixed cells with anti-*L*. *pneumophila* and anti-Flag antibodies, representative images showing the localization of Lug15 on the phagosome (**C**) were acquired with a fluorescence microscope. Bar, 2 µm. Quantitation of LCV-associated 4×Flag-Lug15 is shown in (**D**), and the data are presented as the mean ± standard derivation (SD) of three independent examinations. At least 100 vacuoles were scored for each infection. The data shown in (**A**) and (**B**) are from one representative of three independent experiments.

To determine whether Lug15 is localized to specific organelles in mammalian cells, we transfected HeLa cells with constructs expressing GFP-Lug15 and immunostained the cells with antibodies against proteins specific for several cell compartments. GFP-Lug15 displayed extensive colocalization with the endoplasmic reticulum (ER)-resident protein calnexin (Fig. 4A), suggesting that Lug15 is targeted to the ER. In addition, we performed subcellular fractionation of mCherry-Lug15 expressing cells with sucrose gradient centrifugation, and the data showed that Lug15 coexisted in the fractions containing calnexin (Fig. S7A).

Next, we investigated the intracellular distribution of Lug15 in *L. pneumophila*-infected cells. Owing to the strong nonspecific reaction of our Lug15-specific antibodies in immunostaining, we constructed a plasmid expressing 4×Flag-Lug15 and transformed it into both WT and *dotA^−^* mutant *L. pneumophila*. These bacterial strains were then used to infect U937 cells to determine the cellular distribution of translocated Lug15 by immunostaining with a Flag-specific antibody. Although both strains produced equal amounts of 4×Flag-Lug15 (Fig. 4B), at 2 h post-infection, approximately 65% of vacuoles harboring WT *L. pneumophila* expressing the tagged protein exhibited positive staining for 4×Flag-Lug15 (Fig. 4C and D). However, no signal was detected on vacuoles harboring the *dotA^−^* mutant expressing the tagged protein (Fig. 4C and D). These observations demonstrate that Lug15 is associated with the LCV after being translocated to the host cell by the Dot/Icm apparatus. Additionally, translocated Lug15 cofractionated with the ER marker calnexin as determined by sucrose gradient centrifugation of cells infected with *L. pneumophila* (Fig. S7B).

**Fig 5:**
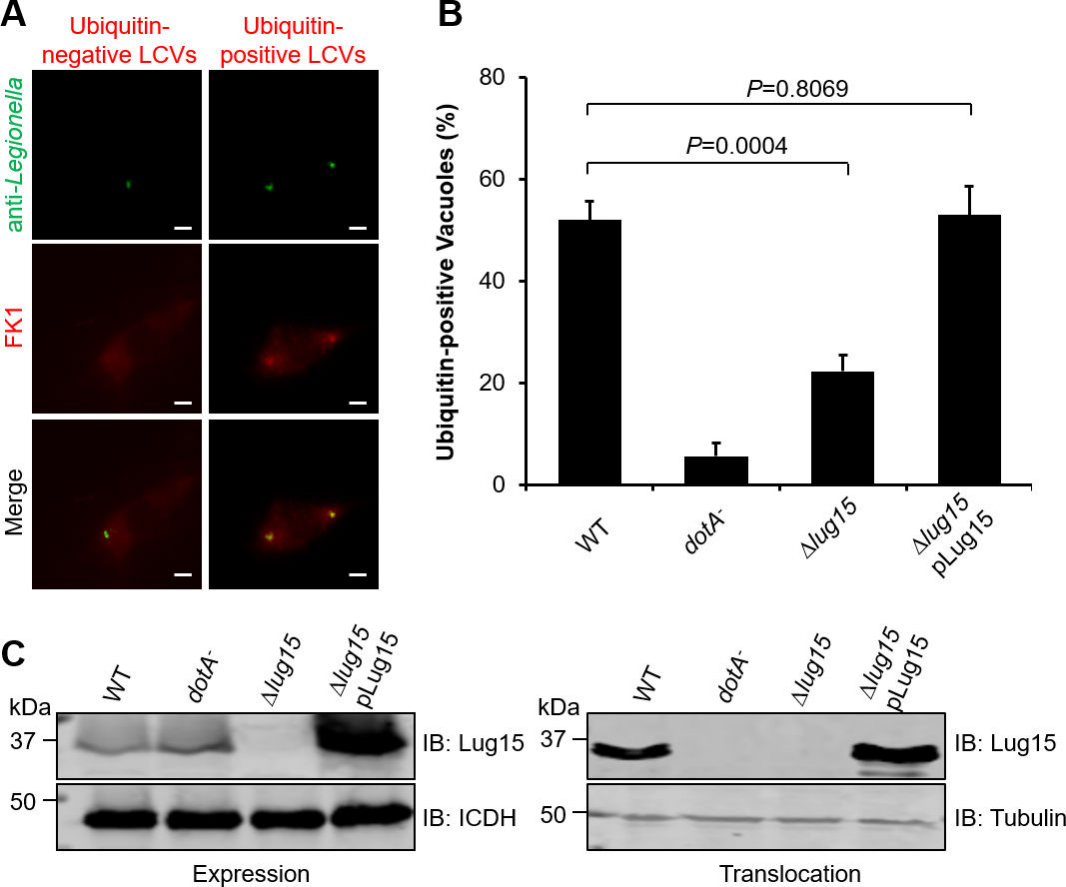
Lug15 impacts the association of polyubiquitin with the LCV. U937 cells were infected for 2 h with the *L. pneumophila* [WT, *dotA*^−^, ∆*lug15*, and ∆*lug15* transformed with a plasmid encoding Lug15 (i.e., pLug15)]. Infected cells were subjected to sequential staining with anti-*Legionella* and anti-FK1 antibodies. Polyubiquitin-decorated vacuoles were visualized by an Olympus IX-83 fluorescence microscope. (**A**) Representative ubiquitin-positive and ubiquitin-negative LCVs. Bar, 2 µm. (**B**) Quantitation of LCVs enriched with polyubiquitinated species. At least 100 LCVs were counted for each infection, and the data shown are the mean ± SD of three independent assays. (**C**) Expression and translocation of Lug15 by relevant *L. pneumophila* strains. Bacterial infection and saponin extraction of translocated proteins were performed as shown in Fig. 4B. Lug15 in the lysates of bacterial cells (left) and infected U937 cells (right) was measured by immunoblotting with an anti-Lug15 antibody. Rabbit anti-isocitrate dehydrogenase (ICDH) and tubulin were included as loading controls for immunoblotting. The data shown in (**B**) and (**C**) are representative of three independent experiments. The statistical analysis in panel (**B**) was performed using unpaired two-tailed Student’s *t*-test; *P* < 0.05 indicates a significant difference.

Dot/Icm-dependent decoration of the LCV by polyubiquitinated species is a typical feature associated with *L. pneumophila* infection. The E3 ligase activity of Lug15 as well as the association of Lug15 with the LCV suggests that Lug15 may affect the enrichment of polyubiquitinated proteins on the phagosome. To examine this possibility, we infected U937 cells with relevant *L. pneumophila* strains for 2 h and immunostained them with an antibody specific for ubiquitinated proteins. In cells infected with WT bacteria, the rate of ubiquitin signal associated with the LCV was approximately 55%; however, in samples infected with the mutant lacking *lug15*, this rate was 22% (Fig. 5). Such defects were fully complemented by a plasmid expressing *lug15* (Fig. 5). These results establish that Lug15 plays a role in the accumulation of ubiquitinated proteins on the LCV.

### Lug15 is involved in the recruitment of Sec22b to the LCV

**Fig 6:**
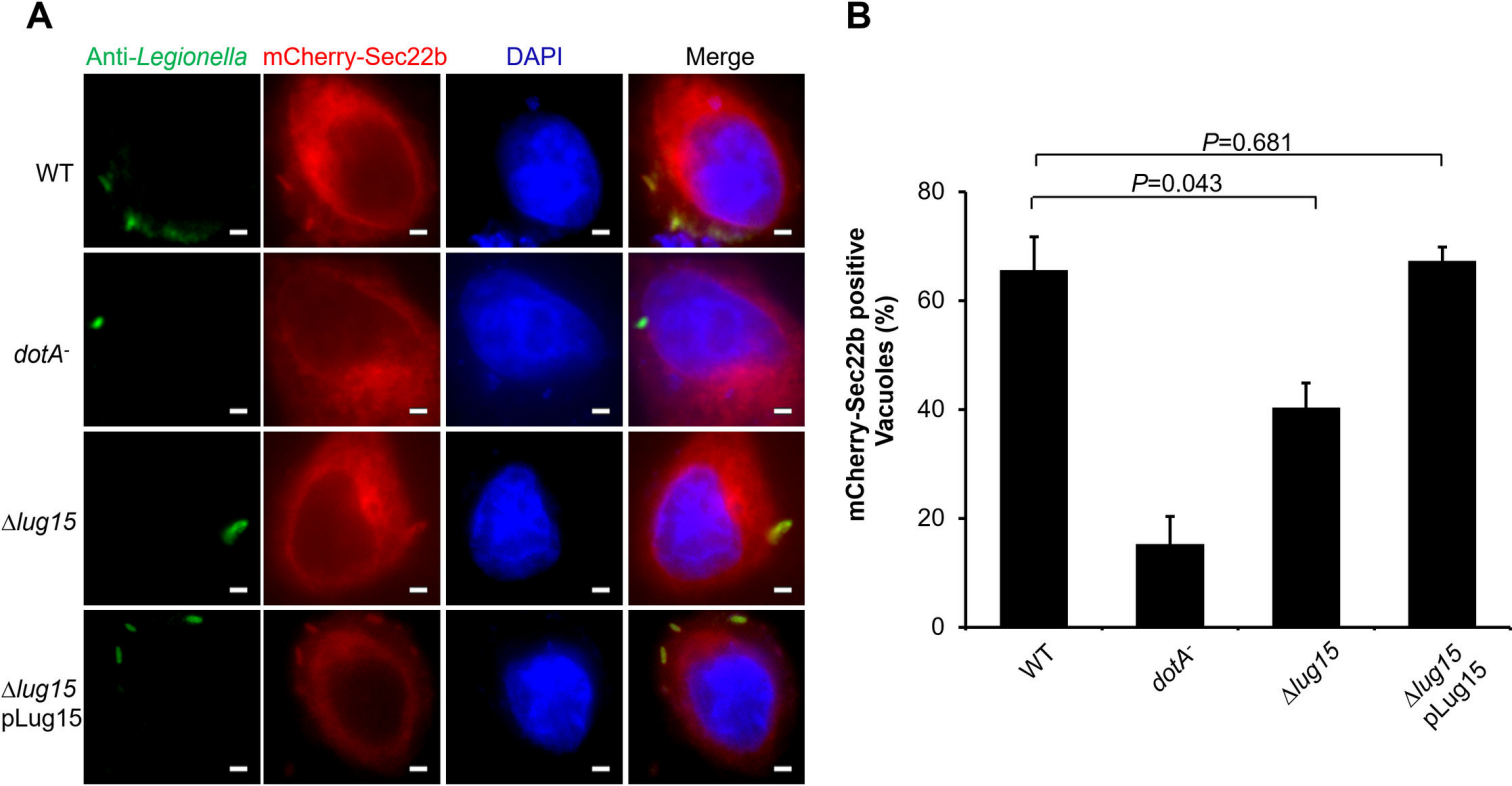
Lug15 plays a role in the recruitment of host Sec22b to the LCV. (**A**) Transiently transfected HeLa cells producing mCherry-Sec22 and FcγRII were infected with opsonized WT, *dotA*^−^, ∆*lug15*, and ∆*lug15* (pLug15) *L. pneumophila* for 2 h at an multiplicity of infection (MOI) of 5. After fixation, the infected cells were stained with an anti-*Legionella* antibody. Nuclei were labeled with DAPI. Fluorescent signals were monitored by an Olympus IX-83 fluorescence microscope. Bar, 2 µm. (**B**) Percentage of LCVs decorated with Sec22b. At least 100 bacterial phagosomes were inspected for each infection, and the values are presented as the mean ± SD of three independent experiments. The statistical analysis in panel (**B**) was carried out through unpaired two-tailed Student’s *t*-test; *P* < 0.05 indicates a significant difference.

At early stages of *L. pneumophila* infection, the ER v-SNARE protein Sec22b is rapidly recruited to the LCV via a Dot/Icm transporter-dependent mechanism (27, 28). Furthermore, a recent study revealed that Sec22b is modified by K63-type ubiquitin chains in cells infected with *L. pneumophila*, and this modification was found to be reversed by the bacterial DUB LotB/Ceg23, an OTU family DUB that specifically cleaves K63-linked polyubiquitin chains (45, 46). However, the effector proteins responsible for Sec22b enrichment and ubiquitination remain elusive. Here, we transiently expressed mCherry-Sec22b in HeLa cells; infected the cells with the WT, *dotA^−^*, Δ*lug15*, or Δ*lug15* (pLug15) strain; and examined the association of mCherry-Sec22b with the LCV. Sec22b colocalization was observed on approximately 65% of LCVs containing WT bacteria at 2 h post-infection (Fig. 6). Strikingly, colocalization was observed on only 40% of vacuoles that contained the ∆*lug15* mutant, a significantly lower percentage than that in samples infected with WT bacteria; in addition, this reduction was rescued expressing Lug15 in the mutant strain from a complementation plasmid (Fig. 6). In addition, we detected a remarkably lower percentage of GFP-KDEL-enriched phagosomes in cells infected with the ∆*lug15* mutant than the WT strain (Fig. S8A), suggesting that Lug15 could affect the recruitment of ER-derived vesicles. As controls, we also stained for reticulon 4B (RTN4B) and Rab1, whose recruitment to the LCV requires the SidE effector family (47) and SidM/DrrA (48, 49), respectively, and found that Lug15 did not detectably impact the association of RTN4B and Rab1 with the bacterial phagosome (Fig. S8B and C), indicating that Lug15 specifically recruits Sec22b to the LCV.

**Fig 7:**
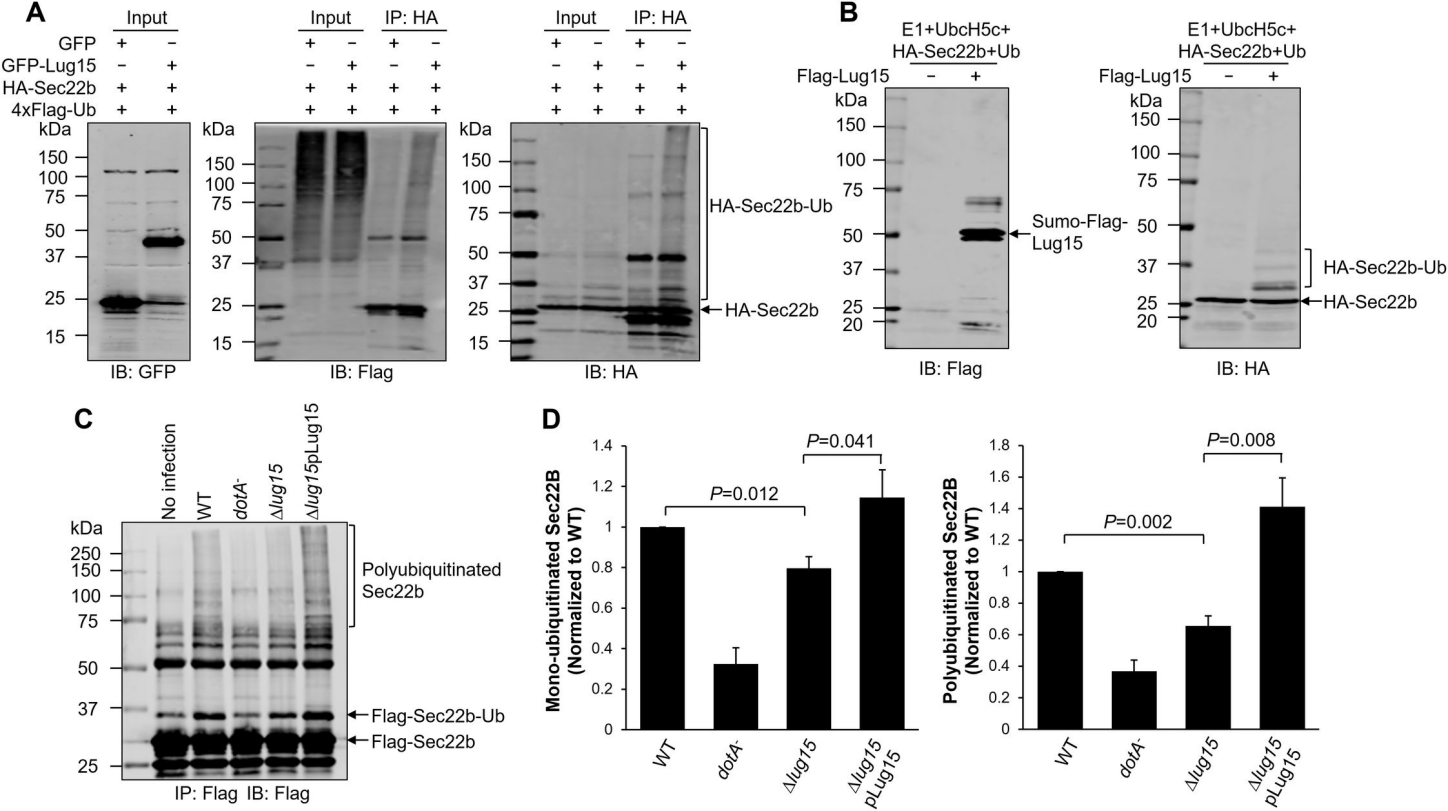
Lug15 catalyzes the ubiquitination of Sec22b. (**A**) Lug15 induces Sec22b polyubiquitination in transfected cells. HEK293T cells were cotransfected with plasmids directing the production of GFP-Lug15, 3×HA-Sec22b, and 4×Flag-Ub. Following immunoprecipitation with anti-HA agarose, ubiquitination of 3×HA-Sec22b was analyzed by immunoblotting with HA- and Flag-specific antibodies. The expression of GFP-Sec22b was confirmed by incubating the cell lysates with an anti-GFP antibody. (**B**) *In vitro* ubiquitination of Sec22b by Lug15. 3×HA-Sec22b transiently expressed in HEK293T cells was precipitated with anti-HA agarose. After elution of the beads with HA peptides, the obtained proteins were added to the ubiquitination reactions in the presence or absence of recombinant Lug15. Ubiquitinated Lug15 and Sec22b in the reactions were detected by immunoblotting with anti-Flag and anti-HA antibodies, respectively. (**C**) Lug15 ubiquitinates Sec22b during *L. pneumophila* infection. HEK293 cells transfected with 4×FLAG-Sec22b and FcγRII were infected with the indicated *L. pneumophila* strains for 2 h at an MOI of 30. 4×FLAG-Sec22b immunoprecipitated with anti-Flag beads from lysates of infected cells was analyzed by SDS-PAGE and immunoblotting with anti-FLAG antibodies. (**D**) Quantification of the monoubiquitinated and polyubiquitinated Sec22b as shown in (**C**) was assessed by ImageJ. Data shown in panels (**A through C**) are representative of three independent experiments. Data shown in (**D**) are the mean ± SD of three independent experiments.

As we have demonstrated that Lug15 is an ubiquitin E3 ligase capable of catalyzing the formation of several distinct polyubiquitin chains, including K63-linked chains, we next tested whether this enzyme can modify Sec22b. First, when HEK293T cells were transfected to coexpress GFP-Lug15 and 3×HA-Sec22b, ubiquitination of Sec22b was detected (Fig. 7A). Second, we transiently expressed 3×HA-Sec22b in HEK293T cells and isolated the protein by immunoprecipitation with agarose beads coated with an HA-specific antibody. A series of reactions containing 3×HA-Sec22b with different combinations of proteins were established. Ubiquitinated Sec22b was detected in reactions containing Lug15 (Fig. 7B), indicating that the SNARE protein is a substrate of this E3 enzyme. Finally, Sec22b ubiquitination occurred in cells challenged with the WT *L. pneumophila* strain but not a strain defective in the Dot/Icm system, consistent with earlier findings (45) (Fig. 7C). Deletion of *lug15* significantly reduced but not abolished the formation of monoubiquitinated and polyubiquitinated Sec22b, and this decrease could be complemented by a plasmid carrying Lug15 (Fig. 7C and D). Taken together, these data suggest that Lug15 is one of the bacterial E3 enzymes that ubiquitinates Sec22b during *L. pneumophila* infection.

### Lug15-induced ubiquitination of Sec22b increases its interaction with the t-SNARE protein Stx3

**Fig 8:**
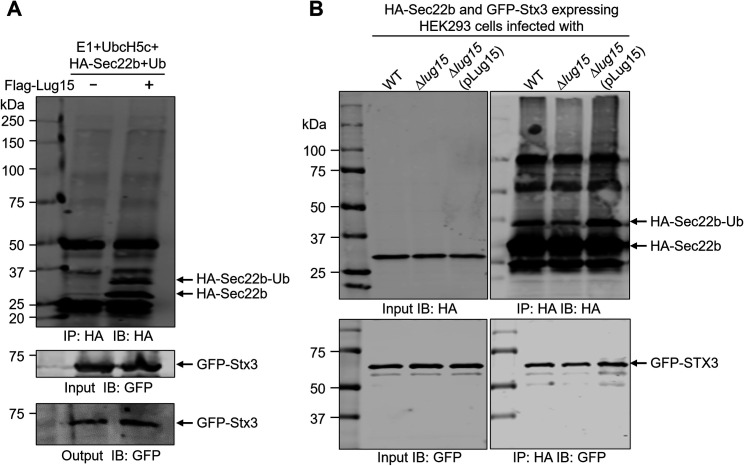
Ubiquitination of Sec22b by Lug15 increases its association with Stx3. (**A**) 3×HA-Sec22b was transiently expressed in HEK293T cells and enriched by anti-HA immunoprecipitation. The HA agarose-bound proteins were then eluted with HA peptides, and aliquots of the eluate were added to the ubiquitination reactions in the presence or absence of recombinant Lug15. After incubation at 37°C for 2 h, 3×HA-Sec22b was immunoprecipitated with anti-HA agarose at 4°C for 2 h. Lysates prepared from HEK293T cells expressing GFP-Stx3 were added to the anti-HA agarose and incubated at 4°C for 2 h. After extensive washing, the beads were resuspended in 1×SDS loading buffer. (**B**) HEK293 cells were transfected to express 3×HA-Sec22b and GFP-Stx3 followed by infection with WT, ∆*lug15*, and ∆*lug15* (pLug15) *L. pneumophila* strains for 2 h. Infected cells were then lysed and subjected to immunoprecipitation with anti-HA agarose. The ubiquitination of Sec22b and its association with Stx3 were detected by immunoblotting with anti-HA and anti-GFP antibodies. The data shown are from one representative of three independent experiments.

*Legionella* infection stimulates noncanonical pairing between Sec22b and PM-derived t-SNAREs, which is critical for the fusion of ER-originated vesicles with the bacterial phagosome (7, 29). In addition, it has been demonstrated that deubiquitination of Sec22b by LotB/Ceg23 during *Legionella* infection causes disassociation of Stx3 from Sec22b on LCVs (45), indicating that the ubiquitination status of Sec22b may be important for promoting this noncanonical pairing. To test this hypothesis, 3×HA-Sec22b purified from transiently expressed HEK293T cells was incubated with recombinant Flag-Lug15 in the presence of ubiquitin, E1, and UbcH5c. After immunoprecipitation with HA agarose, the beads were further incubated with lysates prepared from HEK293T cells transiently expressing GFP-tagged Stx3. We observed a significantly increased association of GFP-Stx3 with Sec22b that reacted with Lug15, ubiquitin, E1, and UbcH5c (Fig. 8A). Moreover, we infected GFP-Stx3 and 3×HA-Sec22b expressing cells with either WT, Δ*lug15*, or Δ*lug15* (pLug15) *L. pneumophila* strains. After immunoprecipitation, the HA-Sec22b-associated GFP-Stx3 was remarkably reduced in cells receiving the Δ*lug15* strain as compared with WT *L. pneumophila* infection (Fig. 8B). Importantly, such reduction can be restored by a plasmid coding for Lug15. Taken together, these results suggest that Lug15-induced Sec22b ubiquitination can promote its noncanonical interaction with Stx3.

## DISCUSSION

Protein ubiquitination is considered one of the most important and versatile PTMs in eukaryotic cells and plays central roles in the regulation of various biological processes (50). Through a sequential multienzyme cascade that involves an E1-activating enzyme, an E2-conjugating enzyme, and an E3 ligase, substrate proteins are covalently modified by different types of ubiquitin chains via isopeptide linkage (50). Ubiquitination determines the fate of a target protein, leading to alterations in its biochemical function, association with interaction partners, organelle targeting, and stability. In eukaryotic cells, the functionality of protein ubiquitination is greatly expanded due to the presence of a large number of E3 ligases, which control both the efficiency and substrate specificity of ubiquitination (38). Because of the critical regulatory roles of ubiquitination in innate immunity, many microbial pathogens have acquired effective strategies to interfere with the host ubiquitin system by the action of virulence factors called effectors (51). Among these, numerous effectors mimic host E3 ligases through the adoption of catalytic domains embedded in eukaryotic RING- or HECT-type E3s (51). For instance, *Salmonella* SopA and *Escherichia coli* NleL are two effectors that mimic eukaryotic HECT-type E3s (52, 53), while AvrPtoB of *Pseudomonas syringae* and NleG encoded by enterohemorrhagic *E. coli* are two examples of RING-like bacterial E3s (54, 55). In addition to the molecular mimicry strategy, some bacterial effectors, represented by IpaH family proteins of *Shigella*, adopt novel E3 ligase folds not found in any eukaryotic E3 (39, 56).

To date, 14 *L. pneumophila* effectors have been experimentally validated to function as E3 ligases, highlighting the importance of exploiting host ubiquitination for successful infection (57). Some of these effectors, including LegU1, LubX, LegAU13/AnkB, and GobX, harbor U- and F-box domains and thus mimic the activity of eukaryotic E3s (57). In contrast, SidC family proteins utilize an E3 ligase fold consisting of a Cys-His-Asp catalytic triad that differs from those of known E3 ligases of eukaryotic or bacterial origin (32). Surprisingly, *L. pneumophila* also possesses effectors that induce substrate ubiquitination by a mechanism chemically different from that of the canonical E1-E2-E3 cascade (57). For example, members of the SidE effector family catalyze phosphoribosyl-linked serine ubiquitination in an E1- and E2-independent manner (16, 17). Instead, they use a two-step process that combines mART and PDE activities to attach ubiquitin to target proteins. The effector MavC catalyzes noncanonical ubiquitination by crosslinking ubiquitin and target proteins via its transglutaminase activity (58). Here, we showed that the Dot/Icm effector Lug15 functions as an E3 ligase that favors E2 proteins of the UbcH5 family to catalyze the formation of multiple polyubiquitin chains with a preference for the K6, K33, K48, and K63 linkage types. Similar to the *L. pneumophila* E3 ligase RavN (59), Lug15 may fold into a structure with remote similarity to eukaryotic E3 domains, although it lacks homology to known E3 ligases based on the primary sequence and secondary structure. Alternatively, Lug15 may use an unrecognized catalytic fold to induce ubiquitination. The primary sequence of Lug15 contains a CxD catalytic motif found in IpaH family E3 ligases, yet mutation of these residues did not result in loss of its E3 activity. Our studies with truncation mutants revealed that a central fragment of Lug15 might be responsible for its function. Further structural study of either the full-length protein or the central fragments would help us to reveal the mechanism of action used by Lug15.

LCVs are decorated with polyubiquitinated conjugates even at late stages of infection, indicating a role in supporting bacterial replication and survival within the vacuole (30). The origin of these polyubiquitinated species has not been fully determined and might involve both host and bacterial factors. One possible route is *de novo* synthesis of polyubiquitin chains by bacterial E3s localized on the LCV, supported by a recent observation that Rab10 is recruited to LCVs and ubiquitinated by SidC/SdcA during *L. pneumophila* infection (34). Consistent with this possibility, *L. pneumophila* E3s, including LegAU13/AnkB and SidC/SdcA, modulate the dynamics of polyubiquitinated proteins associated with the phagosomal membrane (32, 33). Moreover, the *L. pneumophila* genome also codes for effectors possessing DUB activity with distinct chain specificity (6). These E3s and DUBs may coordinate the regulation of the abundance of polyubiquitinated proteins on the LCV at different phases of infection. Indeed, we observed that ubiquitination of Rab10 on the LCV is a net outcome of the interplay between SidC family E3 ligases and the DUB Lem27 (35). Here, we showed that the absence of Lug15 reduces the accumulation of polyubiquitinated proteins on the vacuole, further adding to the complexity of ubiquitin decoration of LCVs by Dot/Icm effectors.

In most cases, bacterial E3s are thought to target host proteins and thereby induce changes in relevant signaling pathways that are beneficial for survival, immune evasion, and intracellular growth. Regardless of the significance of *L. pneumophila* E3s in the exploitation of host ubiquitination for virulence, the host targets of these E3s are mostly unknown. One E3 with verified substrate is the U-box-containing protein LubX, which recognizes the host factor Cdc2-like kinase 1 and directs its polyubiquitination (36). LubX also targets SidH, another Dot/Icm substrate for K48-linked polyubiquitination and subsequent proteasomal degradation to temporally regulate its function (60). Sec22b, the ER-associated v-SNARE protein, has long been known to be recruited to the LCV soon after bacterial infection (27, 28). Such enrichment is required for the subsequent fusion of ER-derived vesicles with phagosomal membranes through noncanonical pairing between Sec22b and PM syntaxins (e.g., Stx3 and Stx4) (7, 29). Sec22b was found to be ubiquitinated upon *L. pneumophila* infection in a Dot/Icm-dependent fashion (45). Although the E3 ligases responsible for such modification are elusive, its ubiquitination is counteracted by LotB/Ceg23, a K63 linkage-specific DUB (45). Our demonstration of ubiquitination of Sec22b by Lug15 established the bacterial E3 responsible for its modification and recruitment to the LCV. Infection of host cells with the Δ*lug15* mutant significantly reduces but not abolishes the level of ubiquitinated Sec22b, suggesting that Sec22b is targeted by multiple *Legionella* E3s including Lug15. LotB/Ceg23-mediated deubiquitination of Sec22b at later stages of *L. pneumophila* infection promotes the dissociation of Stx3 from Sec22b (45). Hence, ubiquitination of Sec22b catalyzed by Lug15 and other E3s might facilitate the noncanonical SNARE pairing between Sec22b and Stx3. In support of this hypothesis, we observed a significant increase in the association of Stx3 with ubiquitinated Sec22b in our biochemical as well as infection experiments. How ubiquitination induced by Lug15 impacts noncanonical SNARE pairing and how it coordinates with LotB/Ceg23 to impose temporal regulation of Sec22b function during bacterial infection require further investigation.

## MATERIALS AND METHODS

### Bacterial strains and plasmids

The bacterial strains, plasmids, and primers used in this study are listed in Tables S1 to S3, respectively. The *E. coli* strains DH5α and DH5αλπ used for molecular cloning were grown in standard LB medium. When needed, antibiotics were used at the following concentrations: kanamycin, 30 µg/mL, and ampicillin, 100 µg/mL. pETSUMO-4×Flag and pZL507−4×Flag were constructed by modification of pETSUMO and pZL507 (61), respectively, with the addition of a 4×Flag tag upstream of the multiple cloning sites. All *L. pneumophila* strains were derivatives of the Lp01 strain (62). *L. pneumophila* was grown and maintained at 37°C on charcoal-yeast extract plates or in ACES-buffered yeast extract (AYE) broth as described (63). The *lug15* (*lpg2327*) deletion strain (Δ*lug15*) was generated by allelic exchange using the suicide vector pSR47S, as described previously (64). Complementation of mutants was accomplished by the introduction of plasmids derived from pZL507 or pZL507−4×Flag that contained the relevant genes. For the expression of proteins in mammalian cells, the gene of interest was inserted into peGFPC1, pcDNA3.1-mCherry, pCMV-4×Flag (65), or pcDNA3.1–3×HA. A QuikChange Site-Directed Mutagenesis Kit (Agilent Technologies) was used to construct Lug15 mutants with single- or double-amino acid substitutions.

### Expression and purification of recombinant proteins

Derivatives of *E. coli* strain BL21 (DE3) harboring plasmids that expressed the proteins of interest were cultured in LB broth supplemented with 30 µg/mL kanamycin at 37°C until the OD_600_ was approximately 0.6–0.8. The cultures were then supplemented with 0.2 mM isopropyl thio-D-galactopyranoside (IPTG) to induce the expression of recombinant proteins and incubated for 16 ~ 18 h at 18°C. Bacteria collected by centrifugation at 4,000 × *g* for 10 min at 4°C were lysed by an ultrahigh-pressure homogenizer (JN-Mini, JNBIO, Guangzhou, China). After clearing cellular debris by centrifugation at 12,000 × *g* for 1 h at 4°C, the soluble fractions were incubated with Ni^2+^-NTA beads to retain recombinant proteins. After extensive washing with lysis buffer containing 20 mM imidazole, proteins were eluted with lysis buffer containing 250 mM imidazole. For purification of glutathione-S-transferase (GST)-tagged proteins, the cleared bacterial lysates were subjected to incubation with Glutathione Sepharose 4B resin (Cytiva) followed by elution with 20 mM reduced glutathione. Purified proteins were dialyzed in buffer containing 25 mM Tris-HCl (pH 7.5), 150 mM NaCl, and 5% glycerol. The protein concentration was determined using the Bradford assay.

### Cell lines, culture method, and transfection

HeLa, HEK293, and HEK293T cells were obtained from the American Type Culture Collection (ATCC) and cultured in Dulbecco’s modified Eagle’s medium (DMEM) (HyClone) supplemented with 10% fetal bovine serum (FBS). The identities of the cell lines were authenticated by short tandem repeat (STR) profiling. All cell lines were regularly examined for mycoplasma contamination by PCR-based tests. BMDMs were prepared from 6- to 10-week-old female A/J mice (Model Animal Research Center of Nanjing University, Nanjing, China) and cultured in RPMI 1640 medium supplemented with 20% L-cell conditioned medium (LCCM) and 20% FBS. All animal procedures were approved by the Institutional Animal Care and Use Committee of Jilin University (approval no. SY201902008). U937 cells were cultured in RPMI 1640 medium supplemented with 10% FBS and were differentiated into macrophages by phorbol 12-myristate 13-acetate (PMA) as described previously (66). All cell lines were grown at 37°C with 5% CO_2_. Transient transfection was carried out when cells were approximately 80% confluent using Lipofectamine 3000 (Thermo Fisher) following the manufacturers’ instructions.

### Bacterial infection

For *L. pneumophila* infection experiments, bacteria were grown in AYE broth () in a shaker (200 rpm) at 37°C to the post-exponential phase, as determined by an OD_600_ of 3.3–3.8 and by bacterial motility monitored under a light microscope. For infection of BMDMs and U937 cells, bacteria were added directly to cells seeded in 24- or 6-well plates. For infection of FcγRII-producing HeLa or HEK293 cells, bacteria were opsonized with an anti-*Legionella* antibody (1:5,000 dilution) produced in rabbits (17) by incubation for 30 min at 37°C prior to being added to cells. For each infection, samples were centrifuged at 1,000 × *g* for 5 min to promote sufficient contact of bacteria with host cells.

Intracellular growth was measured in BMDMs according to a standard procedure described previously (65). In brief, BMDMs were challenged with the tested *L. pneumophila* strains at a multiplicity of infection of 0.05. Two hours after infection, extracellular bacteria were removed by washing the infected samples with prewarmed PBS three times. Then, the samples were refreshed with prewarmed RPMI 1640 medium containing 10% FBS and incubated at 37°C in a CO_2_ incubator. In addition, 2, 24, 48, and 72 h post-infection, saponin was added to the infected samples to a final concentration of 0.02% and incubated at 37°C for 30 min to allow complete cell lysis. Dilutions of the cell lysates were plated onto CYE plates, and the colony-forming units (CFUs) were enumerated after incubation at 37°C for 4–5 days.

To determine the cellular localization of Lug15 and the recruitment of polyubiquitinated proteins during *L. pneumophila* infection, 2 × 10^5^ U937 cells were seeded on glass coverslips in 24-well plates. Infection was carried out with the relevant *L. pneumophila* strains at an MOI of 2 for 2 h. To investigate Sec22b and RTN4B recruitment to the LCV, HeLa cells on coverslips were cotransfected with plasmids expressing FcγRII and mCherry-sec22b or mCherry-RNT4B. Then, 24 h after transfection, the cells were infected with opsonized bacteria at an MOI of 5 for 2 h. Infected U937 cells and HeLa cells were washed with PBS and fixed with 4% paraformaldehyde for 10 min at room temperature before immunostaining with the appropriate antibodies. To detect ubiquitination of Sec22b during *L. pneumophila* infection, HEK293 cells transfected to express FcγRII and 4×Flag-Sec22b were challenged with the relevant bacterial strains at an MOI of 30 for the indicated durations.

### Subcellular fractionation

To determine the intracellular distribution of Lug15, 1 × 10^8^ HEK293 cells were either transfected with mCherry-Lug15 for 24 h or infected with WT *L. pneumophila* at an MOI of 20 for 2 h. The cells were collected by centrifugation at 1,000 rpm for 5 min, and the pellets were resuspended in 2 mL of homogenization solution containing 20 mM HEPES (pH 7.2), 250 mM sucrose, and 0.5 mM EGTA, which was then disrupted with a 7-mL Dounce homogenizer (Wheaton). After removing the unbroken cells and nuclei by centrifugation at 4°C (1,000 rpm for 5 min), the post-nuclear supernatants (PNS) were layered onto a 20%–65% sucrose gradient and ultracentrifuged at 4°C (100,000 × *g* for 1.5 h). Equal volumes of the fractions were collected and analyzed by western blot after SDS-PAGE.

### Immunoprecipitation, western blotting, and immunostaining

Transfected cells were harvested and lysed with RIPA buffer (Thermo Fisher) supplemented with a complete protease inhibitor cocktail (Sigma) for 30 min on ice. Cell debris was removed by centrifugation at 12,000 × *g* for 10 min at 4°C. Then, cleared lysates were mixed with 20 µL of prewashed anti-Flag M2 agarose or anti-HA agarose (Sigma) and incubated at 4°C for 2 h on a rotator. After washing three times with lysis buffer, bead-bound proteins were eluted with 50 µL of 1× sodium dodecyl sulfate-polyacrylamide gel electrophoresis sample buffer or HA peptides (Beyotime, China).

For western blot analysis, protein samples were separated by SDS-PAGE and transferred to nitrocellulose membranes (Pall Life Sciences). After blocking with 5% nonfat milk in PBS for 1 h, the membranes were incubated first with the appropriate primary antibodies and then with IRDye secondary antibodies. Western blot signals were visualized by the Odyssey CLx Imaging System (Li-Cor). The following antibodies were used in the immunoblot experiments: polyclonal antibodies specific for Lug15 generated by immunization of rabbits with recombinant Lug15 following a standard protocol (AbMax Biotechnology Co. Ltd., Beijing, China), rabbit anti-GFP (1:5,000; Sigma, catalog # AB10145), mouse anti-FLAG (1:3,000; Sigma, catalog # F1804), mouse anti-Ub (1:1,000; Santa Cruz, catalog # sc-8017), rabbit anti-Ub-K48 linkage (dilution, 1:1,000, Sigma, catalog # 05–1307), rabbit anti-Ub-K63 linkage (dilution, 1:1,000, Sigma, catalog # 05–1308), rabbit anti-Calnexin (1:1,000, Abcam, catalog # ab22595), mouse anti-tubulin (1:10,000; Developmental Studies Hybridoma BanK [DSHB], E7), rabbit anti-isocitrate dehydrogenase (1:20,000), and mouse anti-HA (1:1000, Sigma, catalog # H3663).

For immunostaining, fixed cells were permeabilized with 0.2% Triton X-100 in PBS for 5 min at room temperature (RT). After blocking with 4% goat serum (Sigma) in PBS for 30 min, the cells were stained with the following antibodies: mouse anti-Flag (1:200; Sigma, catalog # F1804), mouse anti-FK1 antibody (1:1,000; Enzo Life Science, catalog # BML-PW8805), rat anti-*Legionella* (1:2,000), rabbit anti-*Legionella* (1:20,000), and anti-Calnexin (1:100, Abcam, catalog # ab22595). Nuclei were stained with 4′, 6-diamidino-2-phenylindole (Beyotime, China). After staining with appropriate fluorescein isothiocyanate-conjugated or Texas Red-conjugated secondary antibodies, immunofluorescence signals were visualized by an Olympus IX-83 fluorescence microscope.

### *In vitro* ubiquitination assays

For *in vitro* autoubiquitination of Lug15, 0.2 µg of E1, 1 µg of E2, 2 µg of Sumo-4×Flag-Lug15, 5 µg of ubiquitin, 2 mM ATP, and 5 mM Mg^2+^ were added to a 25-µL reaction system containing 50 mM Tris-HCl (pH 7.5) and 1 mM DTT. The reactions were allowed to proceed for 2 h at 37°C. For the dropout assays, a specific component was withdrawn from the ubiquitination reaction. For the time-dependent ubiquitination assay, a 25-µL reaction sample was withdrawn from a 250-µL reaction system at each time point. When indicated, a panel of E2 proteins and ubiquitin K-to-R mutants purchased from R&D Systems was used to determine the E2 and linkage preferences of Lug15, respectively. 3×HA-Sec22b purified from cells transfected with a vector expressing 3×HA-Sec22b was incubated with 2 µg of Sumo-4×Flag-Lug15, 1 µg of UbcH5c, 5 µg of ubiquitin, 2 mM ATP, and 5 mM Mg^2+^ at 37°C for 2 h. All ubiquitination reactions were terminated by the addition of 5×SDS-PAGE sample buffer and incubation at 95°C for 10 min. Protein samples were separated by SDS-PAGE, and ubiquitination was visualized by Coomassie brilliant blue staining or by immunoblotting with the indicated antibodies.

### GST pull-down assay

Ten micrograms of GST or GST-Lug15 was incubated with 20 µL of prewashed GST magnetic beads (Sigma-Aldrich) in a GST binding buffer [50 mM Tris-HCl (pH 7.5), 137 mM NaCl, 13.7 mM KCl] for 4 h at 4°C on an end-to-end rotator. The beads were further washed three times using the GST binding buffer to remove unbound proteins. The beads were then incubated with 10 µg of His_6_-UbcH5c in 1.5 mL of GST binding buffer for 1 h at 4°C on an end-to-end rotator. After extensive washing, the beads were resuspended in 40 µL of 1×SDS loading buffer. Samples were separated by SDS-PAGE and stained by Coomassie brilliant blue.

### Data analysis

Statistical analysis was calculated using unpaired two-tailed Student’s *t*-tests, and the significant difference was set at *P* < 0.05.

## SUPPLEMENTAL MATERIAL

Supplemental material mbio.02382-23-s0001.pdf Fig. S1 to S8 and Tables S1 to S3.

ASM does not own the copyrights to Supplemental Material that may be linked to, or accessed through, an article. The authors have granted ASM a non-exclusive, world-wide license to publish the Supplemental Material files. Please contact the corresponding author directly for reuse.
