# Supplementary material for: Ubiquitination of Sec22b by a novel Legionella pneumophila ubiquitin E3 ligase
Source: mBio. 2023 Oct 26;14(6):e02382-23. doi: 10.1128/mbio.02382-23 (PMC10746214; doi:10.1128/mbio.02382-23)
Supplement: Supplemental material — Fig. S1 to S8 and Tables S1 to S3. [file mbio.02382-23-s0001.pdf]

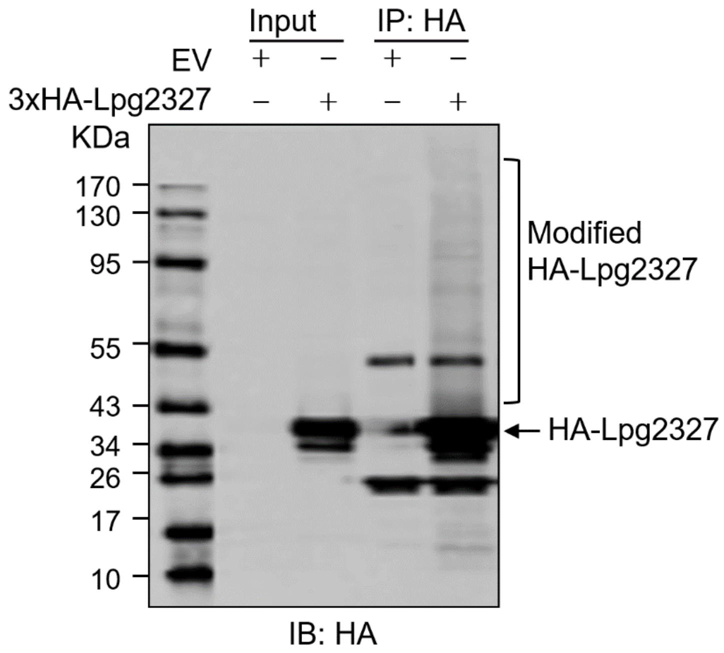

Supplementary Figure 1. Lpg2327 is modified in transiently transfected HEK293T cells. 3×HA-Lpg2327 immunoprecipitated by anti-HA agarose from HEK293T cell lysates was detected by immunoblotting with an anti-HA antibody. The data are from one representative of three independent experiments.

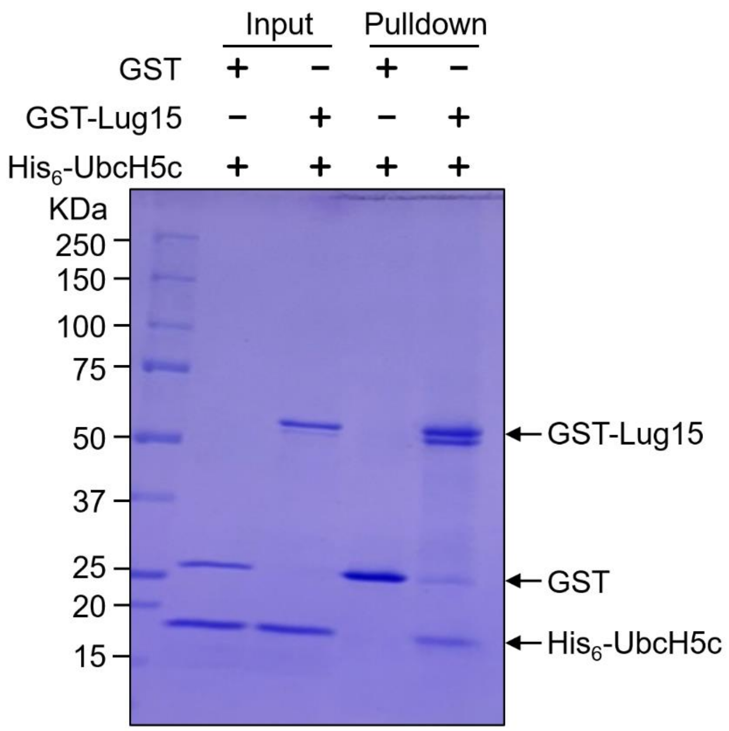

Supplementary Figure 2. Direct interaction between Lug15 and UbcH5c. GST beads coated with GST or GST-Lug15 were incubated with His_6-UbcH5c. After extensive washing of the beads with the GST binding buffer, the beads-bound proteins were separated by SDS-PAGE and detected by CBB staining.

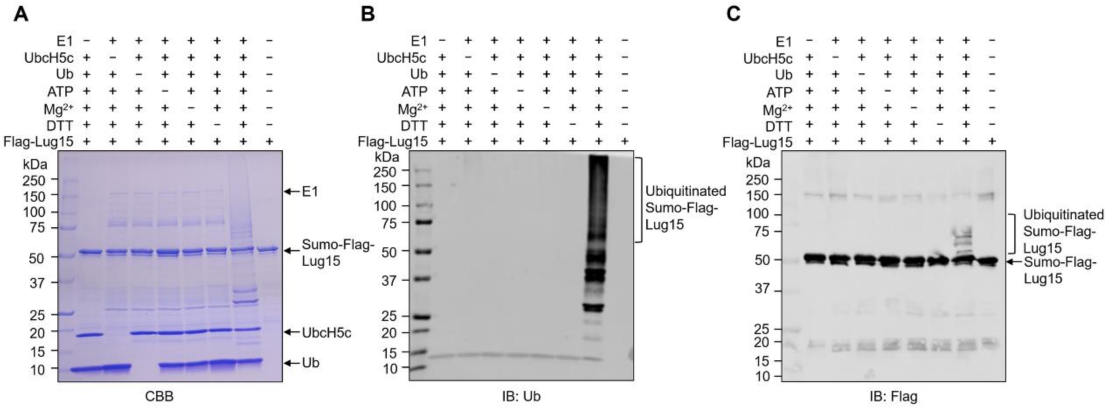

Supplementary Figure 3. The E3 ligase activity of Lug15 requires the canonical ubiquitination machinery. A series of reactions containing the indicated components were set up and allowed to proceed for 2 h at 37 °C. Protein samples separated by SDS-PAGE were subjected to CBB staining (A) or immunoblotting with antibodies specific for Ub (B) and Flag (C). Autoubiquitinated Lug15 was observed only in the sample containing all components of the canonical ubiquitination reaction (Lane 8). The data are from one representative of three independent experiments.

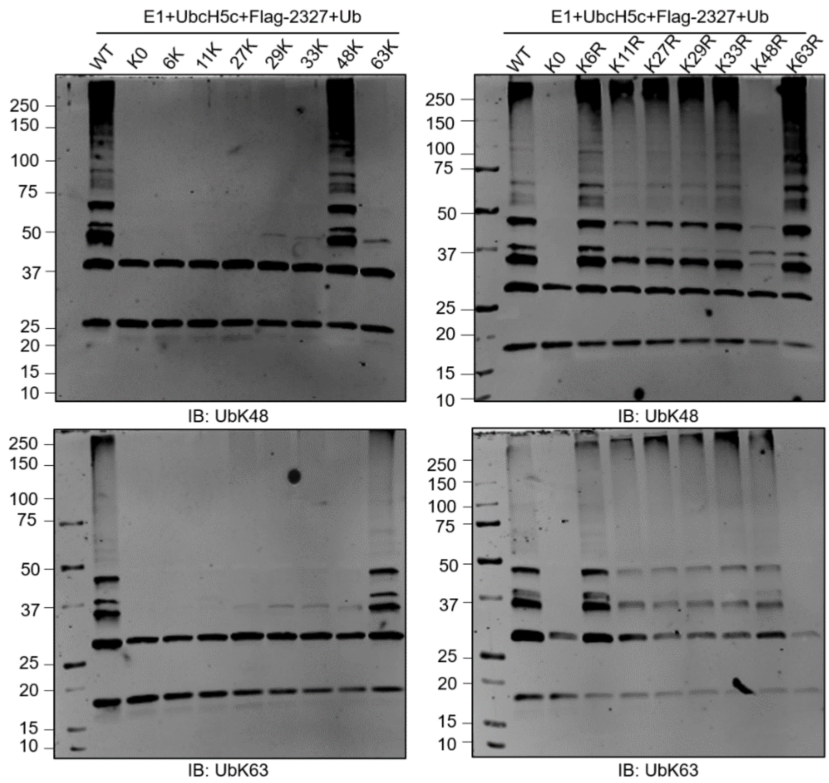

Supplementary Figure 4. Ubiquitin linkage preference of Lug15. Related to Figure 3B. The *in vitro* ubiquitination samples of Figure 3B were further detected by western-blot analysis using K48- (upper) or K63-linkage (lower) specific polyubiquitin antibodies. The data are from one representative of three independent experiments.

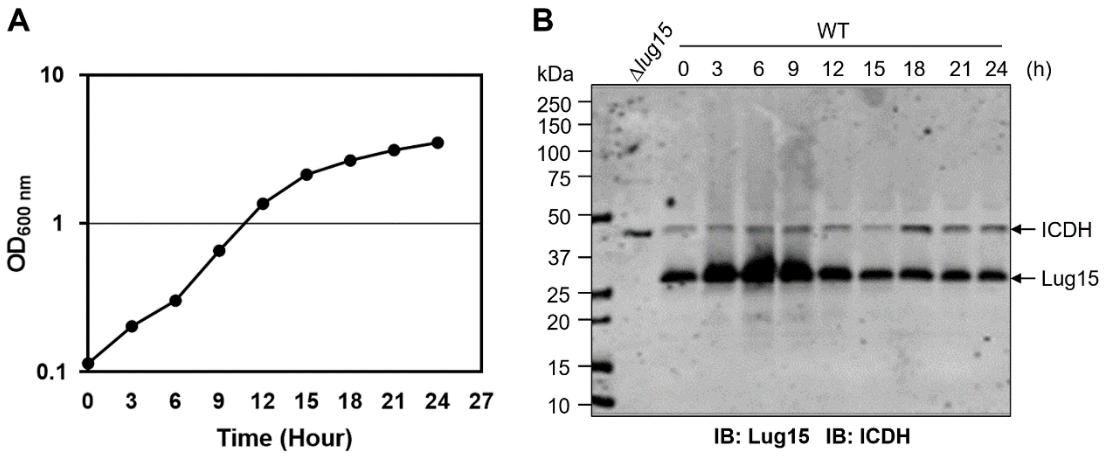

Supplementary Figure 5. Expression profile of Lug15 in *L. pneumophila*. (A) *In vitro* growth of *L. pneumophila*. An overnight *L. pneumophila* culture grown to stationary phase was diluted 1: 20 into fresh AYE broth. Cells were further cultured at 37 °C in a shaker, and bacterial growth was monitored by measuring the OD_{600nm} at 3 h intervals. (B) Lug15 is expressed throughout the growth cycle. Bacterial lysates were prepared from equal amounts of cells harvested at the indicated time points. Total protein was subjected to separation by SDS-PAGE and immunoblotting with a Lug15-specific antibody. The membrane was further probed with an antibody specific for the metabolic protein ICDH to indicate equal protein loading. The Δ*lug15* mutant grown to post-exponential phase was included to identify the specific band of Lug15. The data are from one representative of three independent experiments.

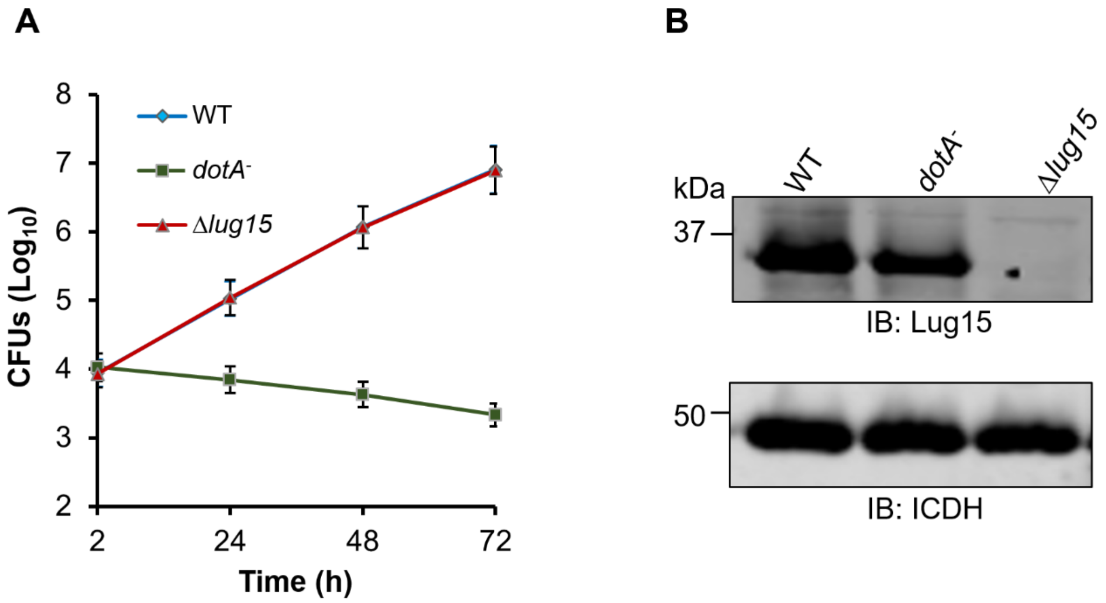

Supplementary Figure 6. Lug15 is dispensable for *L. pneumophila* intracellular growth. (A) The indicated *L. pneumophila* strains were grown to postexponential phase and used to infect BMDMs at an MOI of 0.05. Then, 2, 24, 48, and 72 h post-infection, infected cells were lysed with saponin and plated on CYE plates to allow bacterial growth. CFUs were determined after incubation of the plates at 37 °C for 4 days. (B) Lug15 expression in the *L. pneumophila* strains used for infection was examined by immunoblotting with an anti-Lug15 antibody. ICDH was included as the loading control. The data shown in (A) are representative of three independent assays performed in triplicate.

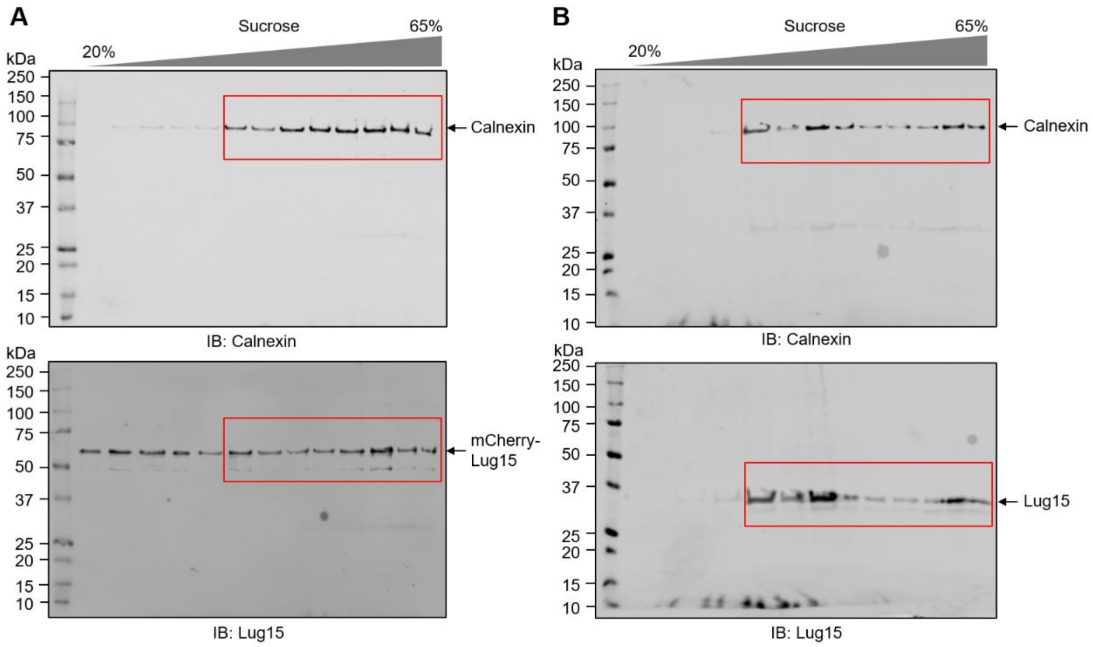

Supplementary Figure 7. Intracellular distribution of Lug15. HEK293 cells were transfected with mCherry-Lug15 for 24 h (A) or infected with WT *L. pneumophila* at an MOI of 20 for 2 h (B). Transfected or infected cells were then lysed and subjected to subcellular fractionation using sucrose density gradient ultracentrifugation. Equal amounts of the fractions were analyzed by western-blot with anti-Calnexin or anti-Lug15 antibodies. The data are one representative of three independent experiments.

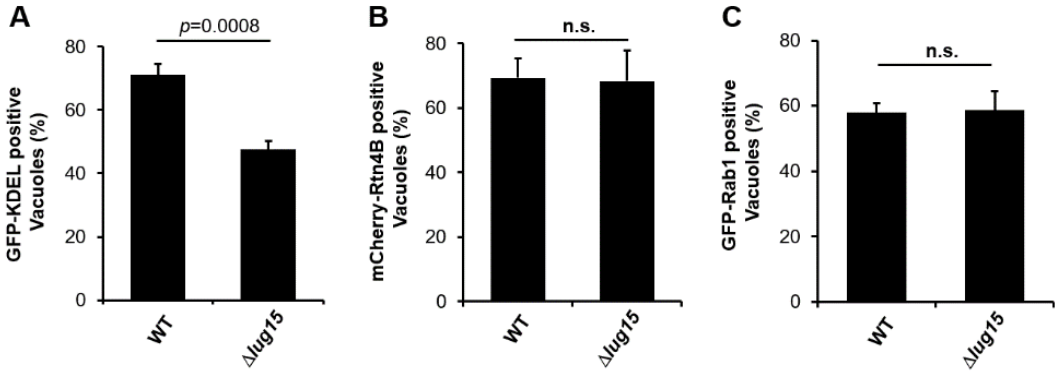

Supplementary Figure 8. Lug15 is important for the recruitment of ER-derived vesicles but not RTN4B and Rab1 to the LCV. HeLa cells were transfected with plasmids expressing GFP-KDEL, mCherry-RTN4B, or GFP-Rab1. Then, 24 h post transfection, the cells were infected with opsonized WT *L. pneumophila* and the Δ*lug15* mutant. After labeling of the intracellular bacteria with an anti-*L. pneumophila* antibody, the association of GFP-KDEL, mCherry-RTN4B, or GFP-Rab1 with the LCVs was measured under a fluorescence microscope. The percentage of LCVs displayed recruitment of GFP-KDEL (A), mCherry-RTN4B (B), or GFP-Rab1 (C) was obtained by monitoring at least 100 vacuoles for each infection, and the values shown are the mean ± SD of three independent experiments. The statistical analysis was conducted by unpaired two-tailed Student's *t* test; $p<0.05$ indicates a significant difference.

Table S1. Bacterial strains used in the study

Strains	Relevant properties	Reference
E. coli		
DH5α (λpir)	supE44 d*lacU169*(φ80*lacZ*Δ*M15*) *hsdR17* *recA1* *endA1 gyrA96 thi-1 relA1 pir tet*::*Mu recA*	Our collection
BL21 (DE3)	F^- *omp*T *hsd*SB (rB^- mB^-) *gal dcm* (DE3)	Our collection
L. pneumophila		
Lp02	Philadelphia-1 *rpsL hsdR thyA*	(Berge & Isberg, 1993)
Lp03	Lp02 *dotA*$^-$	(Berge & Isberg, 1993)
Lp02 (pJB908)	Lp02 + pJB908	(Liu & Luo, 2007)
Lp03 (pJB908)	Lp03 + pJB908	(Liu & Luo, 2007)
KL0001	Lp02 Δ*Lug15*	This study
KL0002	Lp02 Δ*Lug15* + pZL507	This study
KL0003	Lp02 Δ*Lug15* + pZL507-Lug15	This study
KL0004	Lp02 + pZL507-4×Flag-Lug15	This study
KL0005	Lp03 + pZL507-4×Flag-Lug15	This study

References

Berger, K. H., & Isberg, R. R. (1993). Two distinct defects in intracellular growth complemented by a single genetic locus in Legionella pneumophila. Mol Microbiol, 7(1), 7-19.

Liu, Y., & Luo, Z. Q. (2007). The Legionella pneumophila effector SidJ is required for efficient recruitment of endoplasmic reticulum proteins to the bacterial phagosome. Infect Immun, 75(2), 592-603. doi:10.1128/IAI.01278-06

Table S2. Plasmids used in this study

Plasmid	Properties	Reference
pGEX-6P-1	Amp^R, *E. Coli* expression vectors for GST-tagged proteins	GE Healthcare
pGEX-6P-1-Lug15	Full length Lug15 in pGEX-6P-1	This study
pETSUMO	Kan^R, *E. Coli* expression vectors for His-tagged proteins	Novagen
pETSUMO-4×Flag	4×Flag tag before MCS	This study
pETSUMO-4×Flag-Lug15	Full length Lug15 in pETSUMO-4×Flag	This study
pETSUMO-4×Flag-Lug15ΔN100	Lug15ΔN100 (Lug15 residues 100-297) in pETSUMO	This study
pETSUMO-4×Flag-Lug15ΔN120	Lug15ΔN120 (Lug15 residues 120-297) in pETSUMO	This study
pETSUMO-4×Flag-Lug15ΔN140	Lug15ΔN140 (Lug15 residues 140-297) in pETSUMO	This study
pETSUMO-4×Flag-Lug15ΔN160	Lug15ΔN160 (Lug15 residues 160-297) in pETSUMO	This study
pETSUMO-4×Flag-Lug15ΔC40	Lug15ΔC40 (Lug15 residues 1-257) in pETSUMO	This study
pETSUMO-4×Flag-Lug15ΔC60	Lug15ΔC60 (Lug15 residues 1-237) in pETSUMO	This study
pETSUMO-4×Flag-Lug15ΔC80	Lug15ΔC80 (Lug15 residues 1-217) in pETSUMO	This study
pETSUMO-4×Flag-Lug15ΔC120	Lug15ΔC120 (Lug15 residues 1-177) in pETSUMO	This study
pETSUMO-4×Flag-Lug15ΔC140	Lug15ΔC140 (Lug15 residues 1-157) in pETSUMO	This study
pETSUMO-4×Flag-$Lug15_{C224A}$	pETSUMO-4×Flag-Lug15 with mutation C224A	This study
pETSUMO-4×Flag-$Lug15_{D226A}$	pETSUMO-4×Flag -Lug15 with mutation D226A	This study
pETSUMO-4×Flag-$Lug15_{C224AD226A}$	pETSUMO-4×Flag -Lug15 with mutation C224D226A	This study
pETSUMO-4×Flag-$Lug15_{C251A}$	pETSUMO-4×Flag -Lug15 with mutation C251A	This study
pSR47s	R6K suicide vector (Kan^R, sacB)	(Dumenil & Isberg, 2001)
pSR47s-ΔLug15	pSR47s containing the franking region of *Lug15*	This study

pJB908	Amp^R, *thy*$^+$	(Bardill, Miller, & Vogel, 2005)
pZL507	For expression His$_6$-tagged protein *L. pneumophila*	(Xu et al., 2010)
pZL507-4×Flag	4×Flag tag before MCS	This study
pZL507-4×Flag-Lug15	4×Flag-Lug15 in pZL507	This study
pEGFPC1	For expressing N-terminal GFP fusion proteins in mammalian cells	Clontech
pEGFPC1-Lug15	Lug15 in pEGFPC1	This study
pEGFPC1-Syntaxin3	Syntaxin3 in pEGFPC1	This study
pCMV4×Flag	For expressing N-terminal 4×Flag fusion proteins in mammalian cells	(Li et al., 2021)-
pCMV4×Flag-Ub	Ubiquitin in pCMV4×Flag	This study
pCMV-HA	For expressing N-terminal HA fusion proteins in mammalian cells	This study
pCMV-HA-Lug15	Lug15 in pCMV-HA	This study
pCMV-HA-Sec22b	Sec22b in pCMV-HA	This study
mCherry	For expressing N-terminal RFP fusion proteins in mammalian cells	Clontech
mCherry-Sec22b	Sec22b in mCherry	This study
mCherry-RTN4	RTN4 in mCherry	This study
mCherry-Lug15	Lug15 in mCherry	This study
pEGFPC1-Rab1	Rab1 in pEGFPC1	This study
GFP-KDEL	GFP in pEGFPC1 with KDEL signal sequence	This study

References

Bardill, J. P., Miller, J. L., & Vogel, J. P. (2005). IcmS-dependent translocation of SdeA into macrophages by the Legionella pneumophila type IV secretion system. Mol Microbiol, 56(1), 90-103. doi:10.1111/j.1365-2958.2005.04539.x

Dumenil, G., & Isberg, R. R. (2001). The Legionella pneumophila IcmR protein exhibits chaperone activity for IcmQ by preventing its participation in high-molecular-weight complexes. Mol Microbiol, 40(5), 1113-1127.

Xu, L., Shen, X., Bryan, A., Banga, S., Swanson, M. S., & Luo, Z. Q. (2010). Inhibition of host vacuolar H+-ATPase activity by a Legionella pneumophila effector. PLoS Pathog, 6(3), e1000822. doi:10.1371/journal.ppat.1000822

Li, G., Liu, H., Luo, Z. Q., & Qiu, J. (2021) Modulation of phagosome phosphoinositide dynamics by a Legionella phosphoinositide 3-kinase. EMBO Rep 22(3):e51163. doi: 10.15252/embr.202051163.

Table S3. Primers used in the study

Primers	Sequences	Notes
pKM1001	ctgagatctatggcaaaattcaccg	*Lug15* 5F BglII
pKM1002	ctggtcgacttatttgggtgatgaa	*Lug15* 3R SalI
pKM1003	ctggtcgacttaaatcaccgcgttg	*Lug15*$_{(1-257)}$ 3R SalI
pKM1004	ctggtcgacttatttttctaaaaccgg	*Lug15*$_{(1-237)}$ 3R SalI
pKM1005	ctggtcgacttatccagcaagttcctg	*Lug15*$_{(1-217)}$ 3R SalI
pKM1006	ctggtcgacttaatcaactctgtctat	*Lug15*$_{(1-177)}$ 3R SalI
pKM1007	ctggtcgacttattgagttatattttctt	*Lug15*$_{(1-157)}$ 3R SalI
pKM1008	ctgggatccatgttagaagaacaacac	*Lug15*$_{(100-297)}$ 5F BamHII
pKM1009	ctgggatccatggaacaattgaaactg	*Lug15*$_{(120-297)}$ 5F BamHI
pKM1010	ctgggatccatggaagtagaagaaaaa	*Lug15*$_{(140-297)}$ 5F BamHI
pKM1011	ctgggatccatgcagaaagttgatgga	*Lug15*$_{(160-297)}$ 5F BamHI
pKM1012	ctgagatctcgccaaaacgattttt	*Lug15* up BglII knockout
pKM1013	ccaaatcttcttcagcttcagttaaacctttaactggatc	*Lug15* up knockout
pKM1014	gatccagttaaaggtttaactgaagctgaagaagatttgg	*Lug15* down knockout
pKM1015	ctggtcgacgctctgcctgattcaa	*Lug15* SalI knockout
pKM1016	tatttttacggcatcttgagcagctaatttaaactttcttccagcaagttcc	*Lug15* $_{C224A}$ -1
pKM1017	ggaacttgctggaagaaagtttaaattagctgctcaagatgccgtaaaaata	*Lug15* $_{C224A}$ - 2
pKM1018	ttagcttgtcaagctgccgtaaaaata	*Lug15* $_{D226A}$ -1
pKM1019	tatttttacggcagcttgacaagctaa	*Lug15* $_{D226A\ -}$ 2
pKM1020	gtttagctatttttacggcagcttgagcagctaatttaaactttcttccagcaagttcct	*Lug15* $_{C224AD226A}$-1
pKM1021	aggaacttgctggaagaaagtttaaattagctgctcaagctgccgtaaaaatagctaaac	*Lug15* $_{C224AD226A}$-2
pKM1022	cttttaaaggccttgggcaac	*Lug15* $_{C251A}$ -1
pKM1023	gttgcccaaggcctttaaaag	*Lug15* $_{C251A}$ - 2
pKM1024	ctgggatccatggtgttgctaacaa	*Sec22b* 5F BamHI
pKM1025	ctggtcgactcacagccaccagaat	*Sec22b 3R* SalI
pKM1026	ctgggatccatggaagacctggacc	*RTN4* 5F BamHI
pKM1027	ctgctcgagtcattcagctttgcgc	*RTN4* 3R XhoI
pKM1028	ctgggatccatgaaggaccgtctgg	*Syntaxin3* 5F BamHI
pKM1029	ctggtcgacttaattcagcccaacg	*Syntaxin3* 3R SalI
pKM1030	ctgggatccgtgccgctgctgctcggcctcctcggcctggccgtcgccgtgagcaagggcgagg	*GFP-KDEL* 5F BamHI
pKM1031	ctggtcgacttacagctcatccttcttgtacagctcgtcca	*GFP-KDEL* 3R SalI
pKM1032	ctgggatccatgtccagcatgaatc	*Rab1* 5F BamHI
pKM1033	ctggtcgacttagcagcaacctcca	*Rab1 3R* SalI
